# Gastrointestinal Stromal Tumor with Repeated Multiple Cerebral Infarction Mimicking Ovarian Cancer with Trousseau's Syndrome

**DOI:** 10.1155/2022/5537990

**Published:** 2022-04-06

**Authors:** Misa Kobayashi, Yoshirou Otsuki, Hiroharu Kobayashi, Takashi Suzuki, Satoru Nakayama, Hiroshi Adachi

**Affiliations:** ^1^Department of Gynecology, Seirei Hamamatsu General Hospital, 2-12-12 Sumiyoshi, Naka-ku, Hamamatsu, Shizuoka 430-8558, Japan; ^2^Department of Pathology, Seirei Hamamatsu General Hospital, 2-12-12 Sumiyoshi, Naka-ku, Hamamatsu, Shizuoka 430-8558, Japan

## Abstract

We report a case of gastrointestinal stromal tumor (GIST) with repeated multiple cerebral infarctions mimicking ovarian cancer. A 79-year-old postmenopausal woman had multiple cerebral infarctions with a giant pelvic tumor detected by computed tomography. Ovarian cancer with Trousseau's syndrome was suspected. Through laparoscopic biopsy on the tumor surface, she was diagnosed with left ovarian fibrosarcoma; although, the abdominal cavity could not be observed appropriately. Ovarian fibrosarcoma is an extremely rare tumor and still has no adequate treatment strategy. Complete resection was planned. The tumor was extremely fragile, and gelatinous that it easily bled. Meanwhile, the uterus and bilateral ovaries and fallopian tubes were all normal. The tumor invaded only the peritoneum near the left sacral uterine ligament and sigmoid colon, with no peritoneal dissemination. To completely remove the tumor, we performed total hysterectomy with bilateral salpingo-oophorectomy and omentectomy and sigmoidal and rectal resection with colostomy. Despite resuming her anticoagulant therapy on postoperative day 4, she had recurrent multiple strokes. On histopathological examination, tumor showed spindle cell proliferation with severe atypia, increased mitotic activity, and widespread necrosis. Immunohistochemical studies showed positive staining for c-kit, CD34, and DOG1. Thus, she was diagnosed with GIST. This case was rare and highly malignant, with a high risk of recurrence of GIST because of a giant ruptured tumor that had a mitotic activity of 36/10 high-power fields from the sigmoid colon. Multiple cerebral infarctions mimicking ovarian cancer recurred. Therefore, preoperative diagnosis of an atypical GIST was extremely difficult.

## 1. Introduction

Gastrointestinal stromal tumor (GIST) is a rare tumor accounting for only 0.1% and 3% of all newly diagnosed gastrointestinal neoplasms,but is the most common mesenchymal tumor arising in the gastrointestinal tract [1]. Many GIST cases occur under the gastric mucosa and are diagnosed by endoscopic biopsy. However, these tumors can also occur in the lower gastrointestinal tract or other abdominal organs, causing bleeding and necrosis when they grow. Therefore, they need to be differentiated from other diseases. Here, we report a case of GIST with repeated multiple cerebral infarctions mimicking ovarian cancer.

## 2. Case Presentation

A 79-year-old postmenopausal woman (gravida: 3, para: 3) was hospitalized in a different hospital because of bronchial asthma attack but was eventually diagnosed with multiple cerebral infarction. Cerebral infarction developed sparsely in bilateral cerebral and cerebellar hemispheres, possibly caused by embolism. Systemic computed tomography (CT) detected a giant pelvic tumor and deep venous thrombosis in the lower extremities. Ovarian cancer with Trousseau's syndrome was suspected. Hence, 30 mg of edoxaban tosilate hydrate was administered at a time once a day, and 3 weeks after, she had intraperitoneal hemorrhage. Laparoscopic surgery was performed for the hemostasis and pathological diagnosis. Bloody ascites and a giant tumor (appropriately 15 cm wide) occupying the pelvis were noted. The fundus of the uterus and the right ovary were normal, but the left ovary was not observed. No metastases were observed within the visible range of the abdominal cavity. Considering the huge tumor size, rectal adhesion could not be evaluated. Only biopsy on the tumor surface was performed. Histopathological examination revealed fibrosarcoma; thus, she was diagnosed with left ovarian fibrosarcoma. Three weeks after the laparoscopic surgery, she sought for treatment at our hospital.

She had no complaints during her first visit. However, symptoms of dementia such as forgetfulness and low motivation had worsened for approximately 3 months. She had histories of hypertension, diabetes, and dyslipidemia. Regarding family history, her older sister previously had pharyngeal cancer.

**Figure 1:**
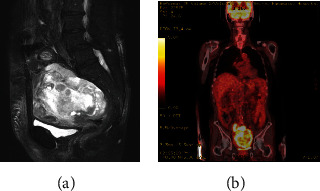
Tumor imaging. (a) Pelvic MRI on sagittal T2WI. A 15 cm tumor showing inhomogeneous high signal intensity (arrow). (b) PET-CT coronal imaging. Increased accumulation of 18F fluorodeoxyglucose in the pelvic tumor.

Pelvic magnetic resonance imaging (MRI) detected an approximately 15 cm pelvic tumor with heterogeneous high signal intensity on T2-weighted images (T2WI) (Figure 1(a)) and high signal intensity on diffusion-weighted imaging (DWI). Positron emission tomography-CT (PET-CT) demonstrated increased 18F fluorodeoxyglucose accumulation in the pelvic tumor (maximum standardized uptake value = 14.6) and no accumulation in other parts (Figure 1(b)). The tumor marker levels such as cancer antigen- (CA-) 125 and CA-19-9 reached 646.0 and 130.2 U/mL, respectively, thereby elevated. The D-dimer level was also elevated (15.0 *μ*g/mL); however, deep vein thrombosis in the lower extremities was not observed. IgG anti-cardiolipin antibodies (13 U/mL), anti-cardiolopin *β*2 -glycoprotein I (<1.2 U/mL), activities of protein S (127%), and protein C (130%) were all normal level. Ovarian fibrosarcoma is extremely rare tumor that still has no adequate treatment strategy. However, the most widely accepted initial treatment is surgical excision [2]. Thus, we planned to perform complete tumor resection.

**Figure 2:**
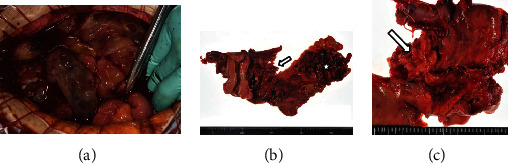
Surgical findings. (a) Extremely fragile, gelatinous, and easily bleeding tumor with bloody ascites. (b, c) The tumor invading only the peritoneum near the left sacral uterine ligament (arrow) and sigmoid colon (asterisk).

**Figure 3:**
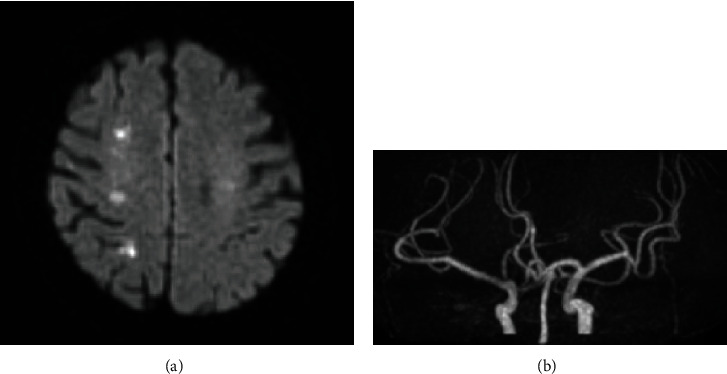
Brain MRI in postoperative day 4. (a) DWI. Multiple high signal intensity in the right cerebrum. (b) Magnetic resonance angiography. No stenosis in the main vessels of the brain.

She was hospitalized 1 week before surgery to manage her anorexia, dehydration, and prerenal nephropathy (creatinine 1.47 mg/dl). Anemia became apparent after infusion (hemoglobin 6.1 g/dl), and bleeding from the pelvic tumor was suspected. Hence, 4 units of red blood cells were transfused, and edoxaban administration was discontinued 3 days preoperatively. In the abdominal cavity, 2000 mL of bloody ascites was collected. The pelvic tumor was extremely fragile and gelatinous and easily bled, and we could easily remove it by hand (Figure 2(a)). After tumor debulking by hand, the uterus and bilateral ovaries and fallopian tubes appeared normal. The tumor invaded only the peritoneum near the left sacral uterine ligament and sigmoid colon, with no peritoneal dissemination. She was then diagnosed with peritoneal fibrosarcoma originating near the uterus or sigmoid colon. To completely remove the tumor, we performed total hysterectomy with bilateral salpingo-oophorectomy and omentectomy and sigmoidal and rectal resection with colostomy (Figures 2(b) and 2(c)). She lost 5147 mL of blood intraoperatively and subsequently received 10 units of red blood cells and 8 units of fresh-frozen plasma. She began taking edoxaban on postoperative day. On postoperative day 4, her consciousness level fluctuated, and brain MRI demonstrated acute multiple right cerebral infarctions with high signal intensity on DWI (Figure 3(a)). MR angiography revealed no stenosis in the main vessels of the brain (Figure 3(b)). The D-dimer level was 1.6 *μ*g/mL at the time of surgery, however, was elevated to 4.2 *μ*g/mL at the onset of cerebral infarctions and continued to be elevated and peaked after a week (23.1 *μ*g/mL). Electrocardiogram showed normal sinus rhythm, and transthoracic echocardiography displayed no intracardiac thrombosis. She had recurrent multiple strokes during anticoagulant therapy, indicating poor neurological prognosis. However, considering her general condition, we avoided aggressive anticoagulant therapy and allowed her to continue her edoxaban therapy. Her consciousness level fluctuated only in a few days and then became clear. She was discharged on postoperative day 22.

**Figure 4:**
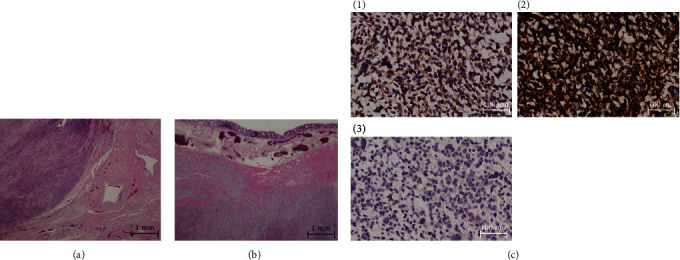
Pathological findings. (a) Posterior caudal side of the uterus (H-E). Tumor infiltration into the uterine serosa. (b) Sigmoid colon (H-E). Tumor infiltration into the proper muscular layer. (c) Tumor immunostaining ((1) c-kit, (2) CD34, (3) DOG1), all positive.

On histopathological examination, tumor showed spindle cell proliferation with severe atypia, increased mitotic activity (36/10 high power fields [HPF]), and widespread necrosis. Tumor cells infiltrated the uterine serosa (Figure 4(a)) and proper muscular layer of the sigmoid colon (Figure 4(b)). Immunohistochemical studies showed positive staining for c-kit, CD34, and DOG1 (Figure 4(c)). Hence, she was diagnosed with GIST.

Given the tumor size, mitotic activity, and tumor localization, the risk of recurrence was high, and we proposed the use of imatinib. However, considering her general condition, she did not receive imatinib therapy. Two months after surgery, she was hospitalized for loss of appetite. She had a large amount of ascites and was diagnosed with pelvic recurrence of GIST. Her general condition deteriorated rapidly, and she died of renal failure in 2 weeks.

## 3. Discussion

GISTs are the most common mesenchymal tumors arising in the gastrointestinal tract and originating from the interstitial cell of Cajal, the pacemaker cell that controls gastrointestinal peristalsis [3]. Histopathologic findings are used to diagnose GIST. GISTs consist of three types: spindle (70%), epithelioid (20%), and mixed (10%). Approximately 95% of GISTs express c-kit, which is the golden standard for diagnosing GIST [4]. Mesenchymal tumors of the uterus and ovaries have different conflicting results in c-kit expression, from less than 5% [5] to more than 50% [6]. For instance, 70% of GISTs express CD34, while 95% express DOG1 [4]. In our case, the tumor was composed of spindle cells and was diffuse positive for c-kit, CD34, and DOG1. Hence, we diagnosed the patient with GIST. However, we have to be careful that sometimes double negativity for c-kit and DOG1 can be seen [7].

The predominant localization of GISTs seems to be the stomach (60%), small intestine (20%-30%), and colorectum (5%-10%). However, GISTs develop rarely in the mesentery, omentum, or retroperitoneum [4]. A case of uterine GIST was also reported [8]. In the present case, the tumor invaded the peritoneum near the left sacral uterine ligament and sigmoid colon. Considering the frequency of occurrence, the tumor might originate from the sigmoid colon. However, the tumor could also originate from the uterus or retroperitoneum. The most recently proposed “modified NIH classification” for the recurrence risk of GIST is defined by the following four factors: number of mitoses, size, location, and rupture [9]. Mitoses of more than 10/50 HPF, a tumor of larger than 10 cm, tumor occurring in areas other than the stomach, or tumor rupture, is an independent high-risk factor of recurrence. This case had a high risk of recurrence because of the 15 cm ruptured tumor with a mitotic activity of 36/10 HPF from the sigmoid colon. Adjuvant imatinib therapy for 3 years in high-risk cases is recommended [10, 11]. However, she did not receive such therapy because of dementia progression with repeated cerebral infarction.

We found some reports about GISTs with a preoperative diagnosis of ovarian cancer [12, 13]. Most of these patients complained of abdominal mass. Images generally showed irregular pelvic masses with necrosis of heterogeneous content ranging from 5 cm to 30 cm in size. The CA-125 levels ranged from 1.6 U/mL to 156 U/mL. Gynecologists typically assume ovarian cancers according to these findings. Almost all of the cases had small-bowel lesions. Conversely, our patient had no complaints. However, MRI detected a 15 cm giant pelvic tumor with heterogeneous high signal intensity on T2WI and DWI, and PET-CT revealed increased 18F fluorodeoxyglucose accumulation in the pelvic tumor. Her CA-125 level was 646.0 U/mL, the highest ever reported. Further, she had multiple cerebral infarctions reminiscent of Trousseau's syndrome associated with ovarian cancer. We strongly assumed she had ovarian cancer.

First described by Armand Trousseau in 1865, Trousseau's syndrome is a migratory superficial thrombophlebitis that might be a forewarning of an occult malignancy. Currently, Trousseau's syndrome is often described as a hypercoagulation disorder in patients with cancer. This syndrome is a spectrum of disorders, ranging at one extreme with thrombosis induced primarily by the production of tissue factor by tumor cells, all the way to a platelet-rich microthrombotic process triggered by carcinoma mucins and involving P- and L-selectins [14]. CA-125 is a valuable surrogate for carcinoma-derived mucin production, and gynecologic cancer is a high-risk factor of cancer-associated deep vein thrombosis [15]. However, Trousseau's syndrome associated with GIST is extremely rare and seldom reported. In this case, she might have common thrombosis because of multiple risk factors such as elderly, hypertension, diabetes, and dyslipidemia. However, multiple cerebral infarctions and deep venous thrombosis in the lower extremities preceded or appear concomitantly with the pelvic tumor. Thus, we could not be denied Trousseau's syndrome. Unfractionated heparin is the first treatment choice of Trousseau's syndrome. Low-molecular-weight heparins are successfully substituted for unfractionated heparin in managing Trousseau's syndrome. Meanwhile, warfarin therapy is inadequate, and novel oral anticoagulants, including edoxaban, still have unknown effects. In this case, convenient edoxaban might be used by former physicians as a thrombus treatment for elderly patients with dementia suffering from cancer. In our case, we continued the administration of edoxaban.

In conclusion, we experienced a rare case of GIST with repeated multiple cerebral infarction mimicking ovarian cancer, and the preoperative diagnosis of this tumor was extremely difficult.
